# mRNA—From COVID-19 Treatment to Cancer Immunotherapy

**DOI:** 10.3390/biomedicines11020308

**Published:** 2023-01-22

**Authors:** Werner Krause

**Affiliations:** Independent Researcher, 13505 Berlin, Germany; w.w.krause@web.de

**Keywords:** mRNA, COVID-19, cancer vaccine, tumor antigens, neoantigens, immunotherapy, clinical applications

## Abstract

This review provides an overview covering mRNA from its use in the COVID-19 pandemic to cancer immunotherapy, starting from the selection of appropriate antigens, tumor-associated and tumor-specific antigens, neoantigens, the basics of optimizing the mRNA molecule in terms of stability, efficacy, and tolerability, choosing the best formulation and the optimal route of administration, to summarizing current clinical trials of mRNA vaccines in tumor therapy.

## 1. Introduction

**Figure 1:**
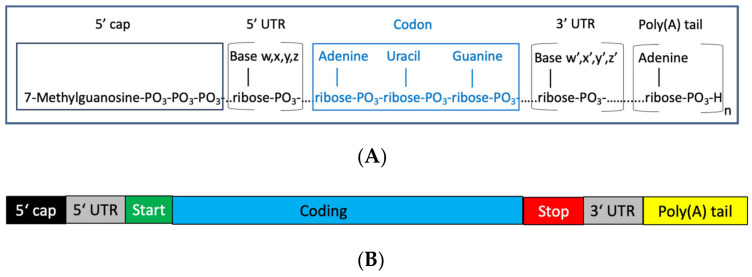
(**A**): General structure of mRNA containing the four nucleobases adenine, cystine, guanine, and uracil, linked to ribose. The resulting nucleosides are connected via phosphate groups. The starting (5′ cap) position is formed by 7-methylguanosine with a three-phosphate moiety as a linker to the first nucleotide. At the end of the molecule, multiple adenosine moieties are attached. A codon comprises three nucleotides, for example, AUG, which is the code for methionine. (**B**): Functional structure of mRNA; details of functions are provided in Table 1. 5′ UTR: 5′ untranslated region. 3′ UTR: 3′ untranslated region. Poly(A)tail: multiple adenosine units.

mRNA—messenger ribonucleic acid—is a single-strand copy of a selected part of the genetic sequence of a gene, negatively charged with secondary and tertiary structure formations. It is produced from DNA and—after appropriate modification—leads to the synthesis of a specific protein by a ribosome. It was first described in 1961 [1]. The feasibility of in vivo expression following the direct injection of in vitro-transcribed mRNA was demonstrated in 1990 [2]. The first commercially available mRNA-based vaccines were Comirnaty (BNT162b2, tozinameran) and Spikevax (mRNA-1273, elasomeran) [3,4]. The major hurdles that had to be overcome before the successful in vivo use of mRNA as a vaccine were instability of the molecule, innate immunogenicity, and delivery. The general structure of mRNA is illustrated in Figure 1. 

mRNA is composed of nucleotide building blocks consisting of a selection of four different nucleobases, adenine, cystine, guanine, and uracil, coupled to ribose which contains a phosphate group as the linker to the next nucleotide. mRNA transfers the genetic information for the synthesis of a specific protein from the genes via the DNA to the protein production facility: the ribosome. The nucleotides constitute the alphabet of the genetic code. Three consecutive nucleotides, for example, AUG, function as codons. Accordingly, by using four letters of the alphabet, A, C, G, and U, 64 different codons are possible. In general, codons define the amino acid which will be recruited next in the protein synthesis. On top of that, a single codon or groups of codons have specific functions, for example, the codon AUG codes for the amino acid methionine and operates as the start codon for the coding part of the mRNA. Table 1 provides an overview of the functionality of codons.

The task mRNA has to fulfil involves the following steps. It starts with the production of a single-strand copy of the genetic code laid down in the DNA, which is performed by RNA polymerase. This process is called transcription and leads to precursor mRNA (pre-mRNA), which—via splicing procedures—finally becomes mature mRNA. In parallel to transcription, the 5′ cap is added to the molecule and the poly(A) tail is attached at the end of the chain. After that, the mRNA is transported from the nucleus of the cell to the cytosol. Various mechanisms can be utilized for this step involving different proteins, such as CBP20 and CBP80. Subsequently, ribosomes take over, read the code, and start the protein synthesis according to the codons provided in the coding section of the mRNA. The synthesis is terminated as soon as a stop codon is reached. Stop codons are UAG (amber), UAA (ochre), and UGA (opal). Thereafter, the mRNA is degraded by ribonucleases. mRNA activates the innate immune response through various RNA sensors such as toll-like receptors (TLRs), retinoic acid-inducible gene I (RIG-I), and protein kinase R (PKR) [5,6,7,8].

## 2. Lessons Learned from COVID-19

The unprecedented speed of the global spread of the COVID-19 pandemic caused by the coronavirus, SARS-CoV2, resulted in an extremely rapid development of mRNA vaccines [9]. In January 2020, the genetic sequence of SARS-CoV-2 was available; in March 2020, the first Phase I trial (NCT04283461) started with an mRNA vaccine (BNT162b2) and a rolling submission procedure was initiated, in which data were provided to the authorities as soon as they were available. In December 2020, BNT162b2 received a temporary emergency use authorization in the UK followed by a conditional marketing authorization (CMA) by the EMA based on data from a global Phase I/II/III study (NCT04368728).

Although SARS viruses are common in humans, vaccines had not been developed since the course of the infection normally was very mild. The SARS outbreak in early 2000 triggered DNA vaccine development [10] but was stopped very soon as the disease vanished on its own. Together with research on MERS-COV, the antigen target rapidly emerged [11]. The spike protein is the major surface protein for most coronaviruses. The virus enters the host cell by endocytosis after binding to angiotensin-converting enzyme 2 [12], followed by the release of the viral DNA. An antibody developed against the spike protein would then inhibit cell entry and thereby neutralize the virus [13]. Furthermore, it could be shown in rhesus monkeys that SARS-CoV-2 infection protects against re-infection [14], mostly via neutralizing antibodies but not via T cell responses [15]. Intramuscularly injected mRNA vaccines mostly induce IgG-type antibodies and less IgA antibodies [16], which provide disease prevention or attenuation but not sterilizing immunity [9]. The preclinical data necessary for the development of a COVID-19 mRNA vaccine were essentially available from the previous SARS and MERS experiments and so saved a lot of development time. In addition to mRNA vaccines, other functional types have been and are in development for the treatment of COVID-19 including DNA and virus vaccines. The latter range from live attenuated virus vaccines via inactivated virus vaccines to recombinant protein vaccines. These types will not be addressed further in this review. For the mRNA-based treatment of COVID-19, various positions of the mRNA molecule had been modified as listed in Table 2.

mRNA capping determines the stability and maturity of the mRNA molecule [41,42]. A eukaryotic translation initiation factor (eIF4E of the eIF4F complex) couples to the 5′ cap and subsequently initiates the translation process [20]. Targets of modification include the 5′ cap [43,44], 5′- and 3′-UTRs, the coding region, and the poly(A) tail [25]. Modification of the 5′- and 3′-UTRs improves the translation and increases the half-life of in vitro transcription mRNA. The 5′-UTR can be rather short containing only three nucleotides [45] or up to several hundreds. The coding sequence is responsible for the target protein that is produced in the ribosomes and determines the rate of mRNA translation and the stability of mRNA in the cytosol [46,47]. The 3′-UTR is vital for the recognition by proteins in the cytosol and also determines the mRNA stability [25,34]. Again, most details are proprietary and have not been disclosed. The addition of a suitable-length poly(A) tail at the 3′ end of mRNA also plays an important role in its successful translation and stability containing ideally more than 90 nucleotides. In some cases, uridine is replaced by pseudouridine, denoted as Ψ. The poly(A) tail can be added to in vitro transcription (IVT) mRNA either through a template vector or by recombinant poly(A) polymerase after the transcription process has occurred [24,25]. 

**Figure 2:**
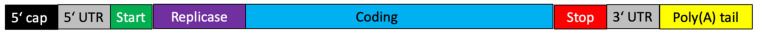
Structure of saRNA containing an additional replicase section.

mRNA which encodes the protein of interest, in the case of SARS-CoV-2, the spike protein, can be administered as such or as self-replicating or self-amplifying mRNA (saRNA) [48,49]. Antigen expression is proportional to the number of conventional mRNA transcripts successfully delivered during vaccination [50], which might result in the need for relatively high vaccine doses. saRNA vaccines are derived from alphaviruses such as Sindbis and Semliki Forest viruses [51,52]. The viral genome is divided into two open reading frames (ORFs): the first ORF encodes for the RNA-dependent RNA polymerase (replicase), and the second ORF encodes the antigen (spike protein) [53,54]. Unlike saRNA, ordinary mRNA is small due to its simpler structure and is characterized by only one ORF. According to Bidram [41], there are three types of saRNA available: plasmid-based DNA saRNA, virus-like particle delivery saRNA, and IVT saRNA [41,55]. Figure 2 provides an illustration of the saRNA design. Due to self-replication, considerably lower doses of vaccines are needed. Cancer mRNA vaccines are mostly non-replicating [56,57]. 

Another alternative is trans-amplifying RNA (taRNA) [58]. In this model, the vector cassette encoding the vaccine antigen originates from the saRNA, from which the replication was deleted to form a trans-replicon. Replicase activity is provided in taRNA by a second molecule, either by a standard saRNA or an optimized non-replicating mRNA (nrRNA), which results in a 10- to 100-fold increase in transcription expression.

mRNA vaccines are different from classic viral vaccines by providing, instead of the antigen itself, the genetic information for producing the antigen. Now, it is up to the host to start protein production. This is a huge benefit compared to administering the antigen to the host. No longer are cell lines required with their potential to produce multiple impurities and the consequence to establish tedious purification and quality control procedures. This is the same issue for DNA vaccines. mRNA vaccines have another significant advantage over DNA vaccines. They have one major hurdle less. DNA needs to enter the cell nucleus [59], while for mRNA vaccines, it is sufficient to reach the cytosol of the cell, which itself is already rather cumbersome.

Vaccine mRNA manufacturing is rapid and cheap by using an IVT from a DNA template with T7 RNA polymerase [60,61]. Since mRNA and appropriate delivery systems are self-adjuvant, they result in strong and long-lasting adaptive immune responses. This is not the case for protein or peptide-based vaccines, which need the addition of adjuvants [62]. Another benefit of mRNA vaccines is that mRNA is much less likely integrated into the host DNA genome than is the case for DNA vaccines [63]. The major reason is that for mRNA manufacturing bacterial fermentation with all its sequelae of isolation and purification is not necessary. Following the expression of antigens, the activity of mRNA is short-lived due to its clearance by RNases, thereby lowering the burden to the host homeostasis. 

mRNA is a large hydrophilic, negatively charged molecule with secondary and tertiary structure formations. The cell membranes are negatively charged as well. Additionally, ion pumps and ion channels maintain a negative potential (−40 to −80 mV) across the cell membrane, keeping the cytosol negatively charged by controlling the balance of most of the essential metal ions (for example, K^+^, Na^+^, Ca^2+^, and Mg^2+^). Naked mRNA is therefore not able to pass this barrier [44]. It was, however, hypothesized that uptake of naked mRNA might occur by endocytosis using cells as mediators. For dendritic cells, this pathway has been described by various groups [64,65,66]. 

Although the mRNA molecule itself is chemically very stable in the dry state, it is rather unstable in the solution, and, moreover, is rapidly degraded by extracellular and intracellular exo- and endo-ribonucleases [23,67,68]. As a consequence, and in order to mask the negative charge, mRNA needs packaging to be able to pass the cell membrane barrier and to protect itself inside the cell cytosol against degradation before it reaches its target, the ribosome. On the other hand, the package needs to be such that the ribosome is still able to detect and then to process the mRNA [69].

Most mRNA vaccines use lipid nanoparticles (LNPs) as the carrier with a particle size between 1 and 100 nm. LNP formulations are composed of an ionizable or cationic lipid, a helper phospholipid, cholesterol or a cholesterol derivative, and a polyethylene glycol (PEG)-modified lipid. The purpose of the ionizable or cationic lipid is an interaction with the negatively charged mRNA. Examples are DLin-MC3-DMA (MC3) [70] or DOPE [71]. The helper phospholipid, for example, 1,2-dioleoyl-*sn*-glycero-3-phosphoethanolamine or DSPC, stabilizes the bilayer structure of the LNP. The pegylated lipid contributes to the stability of the LNP and prevents opsonization followed by uptake in the liver [3,71,72]. However, the PEG group in the LNP formulation is considered to be a possible allergen for anaphylaxis due to approximately 72% of people having some antibodies against PEGs [73]. LNPs are taken up by apolipoprotein or albumin receptor-mediated endocytosis and can electrostatically attach and fuse with the cell membrane using inverted non-bilayer lipid phases [74] or by non-specific pinocytosis [75]. LNPs modified with mannose target DCs through the mannose receptor CD206 [76]. Once inside the cell, LNPs are routed into early endosomes, followed by late endosomes, and finally the lysosomes where the mRNA contents are enzymatically degraded [77,78].

In addition to LNP, other types of formulations such as liposomes [79], ionizable lipids [80], pH-dependent ionizable materials [81], MC3 [70], polymers [82], dendrimers [83], cell-penetrating peptides [84], and other materials have been investigated [44,56,85,86]. Table 3 provides a summary of COVID-19 vaccines with their active ingredients and formulations for vaccines approved in the European Union comprising both mRNA and viral-based vaccines.

The approved SARS-CoV-2 mRNA vaccines use intramuscular (i.m.) administration. During their development, alternative injection routes have been evaluated, for example, intradermal application. A general consensus on what is the best route has not yet been achieved [3]. Although the i.m. administration route induces strong IgG responses that are thought to protect the lower respiratory tract, unlike natural infection it does not drive the secretory IgA responses that are thought to protect the upper respiratory tract [9]. Most vaccines will protect only against infection of the lower respiratory tract and might not induce sterilizing immunity in the upper respiratory tract, which might still enable transmission of the virus. Live attenuated vaccines or viral vectors that can be applied intranasally would probably also lead to a strong mucosal immune response as well as an IgG response. Currently, 68 clinical studies on intranasal administration are listed at www.clintrials.gov. An overview can be found at [87].

Another important issue of mRNA vaccines, which had to be addressed carefully, is manufacturing. A major lesson from the COVID-19 pandemic is that synthesis of the active ingredient is simple because it is cell-free, scalable, and cost effective. Large-scale production of mRNA vaccines consists of a 1- or 2-step in vitro reaction followed by a purification platform with multiple steps that can include DNase digestion, precipitation, chromatography, or tangential flow filtration [88]. A facility dedicated to mRNA production is able to rapidly manufacture vaccines against multiple targets, with minimal adaptation to processes and formulation [61]. Expression may be possible for complex proteins including monoclonal antibodies that are difficult or impossible to generate with conventional expression systems [89]. The manufacturing process comprises the following steps: generation of a plasmid DNA with an RNA polymerase promoter, e.g., T7, and the mRNA sequence of interest, followed by DNA linearization and transcription of the RNA template and by degradation of the DNA. The 5′ cap and the poly(A) tail can be added either during or after transcription. Purification of the mRNA is an important aspect that needs careful attention. For example, various pollutants such as dsRNA in mRNA may activate pattern recognition receptors. HPLC is a suitable method to remove impurities [90,91]. Alternative methods have been described by Shivalingam et al. [92]. Whereas current methods use enzymatic ligation for nucleic acid assembly, their approach is based on the formation of urea and squaramide artificial backbones from minimally modified, commercially available 3′- and 5′-amino oligonucleotides, which provide a one-pot linkage that can be modified on demand for use with stable pre-activated precursor oligonucleotides under reagent-free, mild conditions.

A comparative evaluation of the efficacy in clinical trials of a total of 19 COVID-19 vaccines including mRNA and viral versions has been performed by Fiolet [93]. High efficacy was observed for all the vaccines against SARS-CoV-2. BNT162b2, mRNA-1273, and Sputnik V were superior (>90%) compared to the other vaccines. AZD1222, and CoronaVac were effective in preventing symptomatic COVID-19 and severe infections against the Alpha, Beta, Gamma, or Delta variants. Real-life data revealed effectiveness against the Alpha and Beta variants and reduced efficacy against Delta. A decline was observed for BNT162b2 and AZD1222 after six months, indicating a need for booster vaccinations. In another study on the efficacy of different COVID-19 vaccines, Krammer [9] described the following ranking of neutralizing antibodies elicited by the vaccine candidates: inactivated and AdV5 vaccine candidates < ChAdOx1 nCoV-19 ≈ mRNA vaccines < recombinant protein vaccine candidate [9]. Tolerability was excellent. The inflammatory activity of mRNA vaccines can result in local and systemic inflammation and more autoimmune responses. Serious adverse event rates were rare including myocarditis and pericarditis, cytokine release syndrome, and cerebral venous thrombosis [94,95]. Anaphylaxis was found in 2.5–4.7 cases per million doses, and myocarditis in 3.5 cases per million doses [63]. 

The lessons to learn from the COVID-19 pandemic regarding mRNA vaccines are the following. The pandemic was the strongest driver possible for speeding up the development, production, and distribution of vaccines by providing more than necessary funding options and an immense public pressure to find solutions. Furthermore, mRNA is the ideal candidate for a vaccine since the target antigen is specific for the virus and—so far—has been rather stable regarding evading mutations. If the antigen should slip away, the development of new vaccines is rather straightforward and quick to establish from modifying the mRNA to manufacturing the final product. A still-unresolved issue is the storage temperature, which means freezing, in the case of Comirnaty at −90 °C to −60 °C and for Spikevax at −50 °C to −15 °C during storage and transport, which makes distribution rather cumbersome, in particular in hot areas such as Africa. Freeze-drying of the formulation has been tried as alternative; however, there is a danger of decreasing stability and losing activity [96]. The addition of cryoprotectants, for example, mannitol or sucrose, does preserve the stability and might be a way out of the dilemma [97].

## 3. mRNA-Based Cancer Immunotherapy

The objective of cancer immunotherapy is to manipulate the immune system to effectively eliminate cancer cells [98,99,100]. Immunotherapy mainly targets immune cells. It activates the body’s immune system by inhibiting negative immune regulatory factors and enhancing the ability of immune cells to recognize tumor cell surface antigens to eliminate tumor cells [101,102,103]. Cellular immune responses are mediated by T cells; in particular, CD8^+^ T cells can eliminate tumor cells. Humoral immune activity is mediated by antibodies, which induce clearance by phagocytic cells. On the other hand, the suppressive tumor microenvironment characterized by acidity, hypoxia, and an overexpression of enzymes results in a low immunogenicity of tumor cells and subsequent immunosuppression [104] by preventing T cell infiltration into cancers and causing T cell exhaustion [3]. According to Liu [105], immunosuppressive cells in the microenvironment of tumors include myeloid-derived suppressor cells (MDSCs) [106], tumor-associated macrophages [107], T regulatory cells [108], pro-tumor N2 neutrophils, and cancer-associated fibroblasts. Remodeling the microenvironment, promoting immune cell infiltration, as well as inhibiting tumor angiogenesis and tumor metastasis are imperative for immunotherapy to be effective [109,110,111].

**Figure 3:**
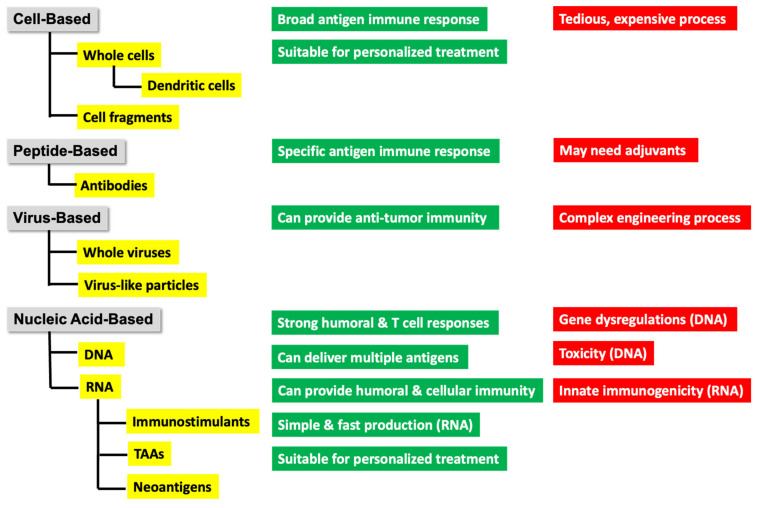
Major types of cancer immunotherapy with their benefits in green and their downsides in red.

The first marketed immunotherapies for cancer were recombinant versions of the cytokine interferon-α (IFNα), which were approved by the FDA in 1986 for hairy cell leukemia [112,113]. Other FDA-approved immunotherapy drugs are monoclonal antibodies functioning as checkpoint inhibitors such as ipilimumab (melanoma), pembrolizumab (various tumors), and nivolumab (various tumors); bispecific antibodies such as blinatumomab (ALL), which is directed against CD19 and CD3; cytokines such as Intron A which is recombinant INF α 2a (hairy cell leukemia, melanoma, follicular lymphoma, and Kaposi sarcoma); engineered T cell therapies, such as tisagenlecleucel (ALL and NHL), which are CD19-specific CAR T cells; oncolytic viruses such as talimogene laherparepvec (melanoma), which is genetically modified HSV type 1 designed to replicate within tumors and produce GM-CSF; and last but not least, the cancer vaccine Sipuleucel-T (prostate cancer), obtained from a strain of *Mycobacterium tuberculosis* [114]. However, the therapeutic effect was far from satisfying [115]. Potential reasons are the low specificity of TAAs, immune escape of cancer cells, and immune suppression in the tumor microenvironment [116]. An overview of therapies is provided in Figure 3.

Cancer immunotherapeutics may be divided into four categories: use of recombinant viruses, tumor and immune cell (mainly dendritic cells)-constructed immunizations, and peptide and nucleic acid-based (DNA or RNA) vaccines [41,98] as illustrated in Figure 3. The term “vaccine” is often used instead of immunotherapeutics. Vaccines can be prophylactic or therapeutic. In the treatment of cancer, both prophylactic and therapeutic treatments have been used. Examples for prophylaxis are HPV (Gardasil-9) [117] leading to cervical cancer and HBV (HEPLISAV-B) leading to hepatocellular carcinoma. The topic of this review is focused on mRNA vaccines for the treatment of cancer. All the other therapies will not be covered, as they have been addressed in various review articles [63,118,119,120,121].

mRNA represents the minimal genetic vector and contains only the elements directly required for the expression of the encoded protein [56]. mRNA vaccines constitute an excellent platform for immunotherapy for a number of reasons [122,123,124]. A major point is the possibility for the simultaneous injection of more than one antigen, resulting in both immune and cell-mediated immunity, thereby increasing the likelihood of tumor tissue eradication [125,126,127].

Antigen selection is the most difficult task in mRNA-based immunotherapy. In the COVID-19 case, the opposite was the case. The choice was easy and straightforward, at least at the beginning when the selected antigen, the spike protein of SARS-CoV-2, was rather stable and more or less exempt from mutations. This changed as soon as the Delta and Gamma variants appeared. In cancer immunotherapy, choices for antigens are tumor-associated antigens (TAAs) [128]. The problem is, however, that these structures are also shared by healthy tissues—although at lower expression levels [129]—and therefore likewise become a target of the mRNA administration leading to autoimmunity and resistance. The ideal antigen should be highly immunogenic, expressed only in cancer cells and not or only at very reduced levels in normal tissue, and it should be essential for cancer cell survival [130]. Tissue-specific antigens (TSAs), therefore, are preferable but much less available. Examples are PSA and HER2. However, immunotherapies based on these TSAs were only of limited success [131]. More preferable options are neoantigens, which originate from non-synonymous mutations in tumor cells and are absent in normal cells [132,133,134,135]. Neoantigens are presented by major histocompatibility complex (MHC) molecules.

Furthermore, mRNA vaccines are not constrained by the patient’s HLA class. mRNA vaccines are preferable over DNA vaccines for several reasons. DNA has to reach the nucleus of the tumor cells in contrast to mRNA, for which it is sufficient to reach the cytosol. For mRNA, there is no danger of splice mutations [64,136]. Modifications addressing efficacy, half-life, etc. are easier to achieve by either modifying the molecule itself, the route of administration, or the formulation [69,137,138,139,140,141].

The mRNA modifications described in Section 2 to improve stability and efficacy apply to cancer vaccines as well. Since uridine-rich sequences activate Toll-like receptors [142], which suppress RNA recognition [32], uridine should be replaced by N1-methyl-pseudouridine (1mΨ), 5-methyluridine, or 2-thiouridine [33]. Other possible modifications include the replacement of cytidine with 5-methylcytidine (m5C) and of adenosine with N1-methyladenosine or N6-methyladenosine. GC-rich mRNA results in several-fold higher transcription efficacy [143] without decreasing the half-life [31]. Further improvements include synthetic analogs of the cap and cap enzymes, which are vital for the stability and maturity of mRNA [42], regulatory elements in the 5′-UTR and 3′-UTR, and the use of poly(A) tails that screen mRNA [24,25,27,31,144,145,146].

Despite all these efforts, the tumor is trying to avoid the effect of the treatment by various methods. Tumor escape mechanisms down-regulate the tumor cell surface antigens which means reducing the immunogenicity and thereby the efficacy of the mRNA vaccine [147]. Another action is the up-regulation of immune checkpoint expression on cell surfaces, for example, PD-L1, which inhibits T lymphocyte activity and induces immune evasion [148]. Furthermore, immunosuppressive cells, MDSCs, and Tregs can be recruited into the tumor microenvironment and cytokines can be secreted which inhibit the immune response [149].

The route of administration of mRNA-based vaccines plays an important role [150]. In addition to intramuscular injection, subcutaneous, intradermal, intranodal [151], and intratumoral routes have been used [152]. Adjuvants such as protamine, granulocyte-macrophage colony-stimulating factor (GM-CSF), and interleukin 2 (IL2) cause strong activation of the innate immune system, which leads to a potent adaptive immune response [153].

mRNA vaccines are not immunogenic, so that multiple administrations are possible [154,155,156]. However, as already mentioned above, there is one caveat. Formulations containing pegylated moieties might still run into the problem of pre-existing antibodies [71,156]. This issue had first been detected in animal models leading to modifications of the formulations [157,158,159] and verification in animals and in patients [160].

According to Barbier [3], the following objectives should be taken into account in the development of an mRNA cancer vaccine. A strong cytotoxic CD8^+^ T cell response is needed to eradicate cancer cells. The antigens should be selected such that they are able to induce highly tumor-specific immune responses. Potential targets are TAAs, TSAs, and neoantigens.

**Figure 4:**
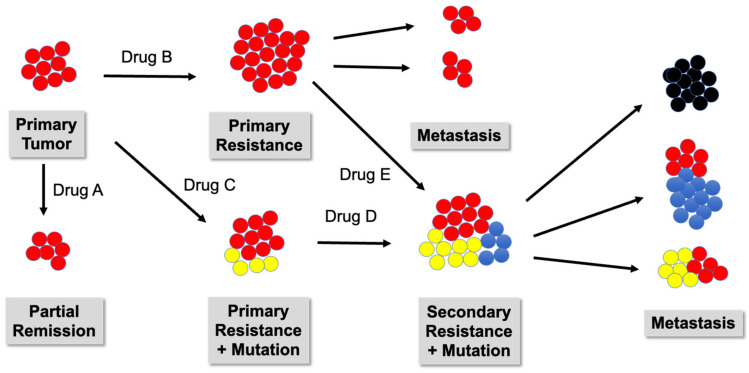
Development of cancer heterogeneity and formation of neoantigens. Treatment with a drug (A, B, C, D, or E) can result in cure, partial remission, or primary resistance that can progress to metastases and/or to mutation resulting in new cancer entities presenting neoantigens. Primary tumor: 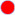. Tumor cells expressing neoantigens A 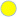, B 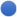, or C 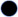.

The major obstacle to this objective is the high variability of antigens across different individuals [161] or even within the same patient. The heterogeneity of tumors—not only between patients but also within the same patient when looking at the time axis—is one of the highest hurdles to treatment success. Most likely, there are not two identical tumors in the whole patient population of a cancer type. This issue is illustrated in Figure 4. A possible way out of this dilemma is the selection of more than one antigen within the mRNA vaccine.

Neoantigens should be the major target whenever possible [135,162]. During carcinogenesis, malignant cells acquire somatic mutations that lead to the production of protein sequences not expressed by normal cells [132,163]. These proteins are called neoantigens. They may be either common across various patients or specific for each patient, which then can be used to develop personalized treatment [164,165,166]. To that end, the tumor is biopsied, and the neoantigens are analyzed by sequencing, encoded in mRNA, and injected into the patient [3,167,168,169]. Common or shared neoantigens include, for example, BRAF and NRAS mutations, which are observed in approx. 50% and 15–25% of melanoma patients, respectively [170,171]. The advantage of this approach is manifold. First, it is an individual treatment, which is a major point taking into consideration the heterogeneity of tumors. Second, mRNA cannot only encode one whole neoantigen but also as many as there are found in the individual patient [3]. Sahin et al. [172] reported that the first injectable mRNA cancer vaccine encoding neoantigens for advanced melanoma patients through intranodal injection achieved potent T cell responses against multiple neoantigens in all patients after vaccination. However, although personalized cancer vaccines based on neoantigens have shown encouraging results, a large number of predicted neoantigens tend to trigger very few actual anti-tumor responses [173]. Table 4 provides a selection of neoantigens.

Dosing of mRNA can be achieved by titrating up or down, depending on the need, weight, and disease state of the patient. The duration of action is intrinsically limited by mRNA degradation, reducing the likelihood of irreversible side effects, and enabling the treatment of acute indications [184,185].

Formulations for use in cancer treatment are similar to those described for COVID-19 administration. However, as opposed to the delivery systems of mRNA vaccines for pathogen infections, therapeutic mRNA vaccines for cancer treatments are required to generate both robust CD8^+^ and CD4^+^ T cell responses [186]. The activation of type I interferon (IFN) proved important in developing a cytotoxic T cell response [187]. Early on, naked mRNA dissolved in Ringer’s solution at a concentration of 1.0 mg mL^−1^ was injected into separate inguinal lymph nodes in thirteen melanoma patients [172]. However, since naked mRNA cannot enter cell membranes freely but only by cell-mediated endocytic pathways [56,64,65,66], preferred formulations not only for the treatment of COVID-19 but also for cancer therapy are LNPs which can substantially affect intracellular delivery efficiency, determine cell specificity of delivery, and modulate immunogenicity [3]. Ionizable lipid components of LNPs play a key role in multiple aspects of mRNA delivery, including particle formation, cellular uptake, and endosomal escape [44,57,188]. Their self-adjuvant activity, resulting in the stimulation of specific parts of the immune system, is an important aspect of mRNA LNPs. Examples are the stimulator of IFN-γ (STING) pathway and the TLR–RIG-I-like receptor (RLR)-independent mediator of innate immune responses [3,189]. Other formulations that have been developed are hybrid lipopolymer shell mRNA nanoparticles or lipoplexes [190]; nanocapsules with flexible polysaccharide shells and hollow cores, termed a sugar-capsule composed of mannan carrying mRNA [191]; and a nanoparticle platform, called mRNA Galsomes [192]. Reviews on formulations are available from various groups [193,194,195,196]. Large-scale production has been established extremely rapidly [88]. Table 5 provides a selection of mRNA vaccines in development.

As can be seen from Table 5, the range of indications is rather broad reaching from head and neck, breast, lung, pancreas, prostate, gastric, and rectal cancer to melanoma. BNT111 encoding the antigens, tyrosinase, NY-ESO-1, MAGE A3, and TPTE, is currently being investigated in melanoma. A Phase I trial (Lipo-MERIT) using liposomal mRNA, injected intravenously, in combination with or without the checkpoint inhibitor PD1, showed in an interim analysis induction of strong CD4 and CD8 T cell immunity against the vaccine antigens [197]. A Phase II trial with BNT111 ± cemiplimab is ongoing. Preliminary data from a Phase I/II trial of liposomal BNT112 encoding the five prostate cancer-specific antigens, kallikrein-2, kallikrein-3, acid phosphatase prostate, HOXB13, and NK3 homeobox 1, as monotherapy or in combination with cemiplimab, in metastatic castration resistant prostate cancer (mCRPC) showed that all five antigens were immunogenic and responses to each antigen were observed in at least two patients [198]. BNT121 was studied in melanoma. Thirteen metastatic patients received repeat administrations in inguinal lymph nodes showing clear immunological responses and some evidence of clinical activity [3]. Data from a Phase I trial in solid tumors with nine i.v. injections of BNT122 (RO7198457) encoding twenty patient-specific antigens have been reported by Braiteh [199]. BNT122 induced the pulsatile release of pro-inflammatory cytokines with each dose, consistent with the innate immune agonist activity of the RNA. Neoantigen-specific T cell responses were observed in peripheral blood in 14/16 patients (87%). Overall, 1 out of 26 patients had a complete response and 11 had a stable disease. mRNA-2416 is encoding the immune checkpoint modulator, OX40L, and is administered intratumorally. In a first trial with 41 patients with different malignancies, the compound did not meet the response criteria for solid tumors. A Phase II study in ovarian cancer in combination with durvalumab (NCT03323398) is ongoing [3]. mRNA-2752 encoding OX40L, IL-23, and IL-36γ was investigated in colorectal cancer (NCT03739931). However, in 17 patients there were no responses [198]. mRNA-4157 is a personalized mRNA encoding up to 34 neoantigens. In a Phase II solid tumor study, either as monotherapy (*N* = 16) or in combination with pembrolizumab (*N* = 63), multiple disease-free patients were observed during the study [199]. The safety and immunogenicity of mRNA-4650 encoding-defined neoantigens, mutations in driver genes, and HLA-I–predicted epitopes were determined in patients with metastatic gastrointestinal cancer (NCT03480152). CD8^+^ and CD4^+^ neoantigen-specific T cells elicited by the vaccine could be detected. However, since in 3 of 4 patients, no clinical response was observed, Phase II was not initiated [205]. TriMix, a mixture of monocyte-derived dendritic cells electroporated with mRNA encoding CD70, CD40 ligand, and constitutively active TLR4, as well as the tumor-associated antigens tyrosinase, gp100, MAGE-A3, or MAGE-C2 were administered together with ipilimumab in patients with advanced melanoma [206]. Enzyme-linked immunospot assay responses detected after in vitro T cell stimulation were shown in 12/15 patients. Vaccination in combination with ipilimumab resulted in robust CD8^+^ T cell responses in a meaningful portion of late-stage melanoma patients, and obviously in patients with a clinical response. TriMixDC-MEL, autologous monocyte-derived mRNA co-electroporated dendritic cells with mRNA encoding CD40L, CD70, and caTLR4, was administered i.v. to 21 late-stage melanoma patients. The control group without treatment comprised 20 patients. One year after randomization, 71% of patients in the study arm were alive and free of disease compared to 35% in the control arm [208]. The median time to non-salvageable recurrence was superior in the TriMixDC-MEL arm (median 8 months (range 1–6) vs. not reached. CV9103 (RNActive^®^) is based on four prostate-specific antigens, PSA, PSMA, PSCA, and STEAP and was administered intradermally in a Phase I trial in prostate cancer patients [210]. In a subsequent Phase II study, antigen-specific T cells were detected in around 80% of prostate carcinoma patients independent of their HLA background. A majority of immune responders, around 58%, reacted against multiple antigens, and the responses were detected against all antigens independent of their cellular localization. Individual patients were showing prolonged stabilization of PSA levels after initial rises. One patient had a greater than 85% drop in his PSA level [211]. CV9104 is a mixture of six mRNAs, each encoding one antigen, PSA, PSCA, PSMA, STEAP1, PAP, or MUC1. No significant difference in OS was found between the vaccine and control arm. There were also no significant differences in the rPFS endpoints and time to symptom progression [212]. CV9201 is an RNActive^®^-based cancer immunotherapy encoding five NSCLC antigens, NY-ESO-1, MAGE C1, MAGE C2, survivin, and TBG. In Phase IIa, antigen-specific immune responses against ≥1 antigen were detected in 63% of evaluable patients after treatment. The frequency of activated IgD CD38 B cells increased >2-fold. In total, 31% of evaluable patients in Phase IIa had a stable disease and 69% had a progressive disease [213]. MEDI1191, which is encoding IL-12, showed in a Phase I study in combination with durvalumab two partial responses in patients who had received prior immunotherapy and also had PD-L1-negative tumors [217]. The direct injection in 15 melanoma patients of naked mRNA encoding various antigens has been described by Weide et al. [218]. An increase in the anti-tumor humoral immune response was seen in some patients. However, a demonstration of the clinical effectiveness of the direct injection of copy mRNA for anti-tumor immunotherapy was not shown. Another Phase I trial in 30 renal cell cancer patients using naked mRNA coding for MUC1, CEA, Her-2/neu, telomerase, survivin, and MAGE-A1 has been published by Rittig et al. [219]. The induction of CD4^+^ and CD8^+^ T cell responses was shown for several TAAs. In summary, the clinical efficacy of mRNA vaccines looks like a mixed bag of different outcomes ranging from no effect to a rather good response, which means that the route to sufficient efficacy still is rather steep but, nevertheless, nourishes high hopes for the future.

## 4. Conclusions

The tremendous success and unbelievable speed in developing and bringing to the market COVID-19 mRNA vaccines has raised high expectations for duplicating this performance in other indications, in particular in cancer therapy. mRNA vaccines are easy to design and can rapidly be modified if there is a need for change, exhibiting an extremely broad versatility of building blocks, structural elements, and formulations of the synthetic mRNA including the targeting of defined cells, duration of expression, and immunological effects. Dosing can be adjusted according to the individual needs of the patients, and there is the possibility to individualize treatment by adjusting the selection of antigen(s) to the specific tumor type of each patient. However, the hurdles in cancer immunotherapy are much higher than in fighting coronaviruses. In particular, the following issues need to be addressed: neoantigen use, LNP modifications, combination treatments, immune escape of cancer, and the therapeutic vs. prophylactic use of mRNA vaccines.

If available, neoantigen-specific vaccines, which are currently considered a top priority in cancer immunotherapy, result in a potential killing of all cells exhibiting the neoantigen epitope but leave any other cancer cells unharmed (which might then start or continue multiplying). As a consequence, the cancer genotype and phenotype changes, and the tumor will continue growing [220]. Targeting multiple neoantigens within a single vaccine and/or combining the vaccine with non-mRNA tumor therapies should be targeted to reduce immune evasion and effectively eliminate tumors. Similarly, the general mechanisms for the immune escape of cancer, which are much more complex than those for breakthrough infections of pathogens [221], need to be addressed. Combinations of mRNA vaccines with agents that can reverse immunosuppression such as immune agonists or cytokines and compounds that block immune checkpoints have been shown to be more potent than a single administration of vaccine therapy [41,64,214,222,223]. However, not all patients are responsive to these treatments [224]. Nevertheless, this approach, and the combination of one or more non-mRNA agents fighting immune escape with multitarget mRNAs, should be continued even more extensively in the future.

LNPs are state-of-the-art for mRNA vaccines. However, they bear several pitfalls. Since they are particles, they can be taken up by macrophages of the liver or the spleen—irrespective of their administration route—thereby reducing the activity at the intended site of action, the tumor, and/or leading to side effects at off-target accumulation sites. The side effects might include toxicity and/or immunogenicity [150,225] and inflammation exacerbation [226]. The effects depend on various factors including the constituents of the vaccine carrier and the antigen. For example, cationic/ionizable lipids can induce inflammation by activating TLR pathways and lead to cell toxicity. On the other hand, this inflammatory effect is a useful contributor to the adjuvant activity of the LNP. Accordingly, the positive adjuvant activity and the negative inflammatory action need to be carefully balanced by selecting an appropriate cationic/ionizable lipid [227]. Other strategies have been described including the effort to make the particles invisible to macrophages by modifying their surface with, for example, PEG derivatives. The caveat for this solution has been illustrated above: the potential immunogenicity of PEG compounds [160]. Yet another possibility has been published by Dirisala [228], regarding the in situ stealth coating of liver sinusoids using linear or two-armed PEG–conjugated oligo(L-lysine). Another approach could be the reduction in the size of the particles. Small particles are no longer taken up by the liver or the spleen; they keep circulating in the blood, and therefore prolong the activity, as has been described for blood-pool contrast agents [229]. In summary, the mRNA vaccine carrier for cancer immunotherapy, in particular LNPs, needs further consideration to optimize activity and tolerability.

A further issue that needs to be addressed is the observation that vaccine treatment may not successfully reach the tumor site, in particular solid tumors [175]. Probably, cancer vaccine treatments are more suitable for patients with an undisturbed immune system, a relatively small tumor load, and a greater risk of recurrence.

The focus in cancer immunotherapy should also be directed towards prophylactic vaccination, which has already been very successful in HPV and HBV leading to cervical cancer and hepatocellular carcinoma. An extension to other tumor types seems mandatory.

## Figures and Tables

**Table 1:** Building blocks of mRNA and their functions. The Kozak sequence is a group of nucleotides that initiates the protein translation in the ribosome. An example is 5′-GCCGCCRCC**AUG**G-3′, where red color indicates that these nucleotides are fixed, **AUG** is the start codon encoding for methionine, R stands for A or G, and the function of GCC is not well defined.

Component	Nucleotides	Function
5′ cap	7-Methyl-G	Essential for ribosome recognition, transcription, protection against ribonucleases
5′ UTR	A, C, G, U; from 3 to several hundred nucleotides	Contributes to stability, localization, and translation efficiency Normally proprietary knowledge
Start	AUG within a Kozak sequence	Codes for methione and initiates translation
Coding	A, C, G, U	Regulation of splicing Decoding (reading) by ribosomes and translation into the target protein
Stop	UAG: amber UAA: ochre UGA: opal	Terminates the translation process
3′ UTR		Contributes to stability, localization, and translation efficiency, potentially involved in disease susecptibility
Poly(A) tail	A	Protects against exonucleases, aids in transport from nucleus to cytosol and in translation

**Table 2:** Possible modifications of the mRNA molecule for use as COVID-19 vaccines with the spike protein of SARS-CoV-2 as target antigen. Most modifications used for approved vaccines are proprietary and therefore not publicly available.

Position	Modification	Effect	Reference
5′ cap	Methylation of the first nucleotide at position 20 (cap1 structure) CleanCap system: 5′ cap + 2′ methylated adenosine followed by guanosine	Allows incorporation of cap1 at the 5′ end of any mRNA and does not limit the concentration of any of the four nucleotides	[17,18,19,20]
5′ UTR	Apply 5′ cap in multiple versions Incorporate synthetic cap or anti-reverse cap analogues Strong Kozak translation signal Avoid the presence of start codon (AUG) and non-canonical start codons (CUG) Use shorter 5′ UTR Remove highly stable secondary structures Avoid hairpin loops	Increases stability and translation resulting in higher efficiency and longer half-life Increases protein synthesis during fibroblast conversion to induced pluripotent stem cells Improves translation	[21,22,23,24,25,26,27,28,29]
Start			
Coding	Replace a nucleotide with N1-methyl-pseudouridine (N1mΨ) High GC sequence better than a low GC sequence Replace cytidine with 5-methylcytidine (m5C) or uridine with pseudouridine (Ψ) or 1-methylpseudouridine (m1Ψ) To be carefully optimized since it can affect the rate of translation	Better base pair stability and mRNA translation 100-fold higher translation Reduces innate immune activation The rate of translation needs to consider the formation of the tertiary structure of the protein	[30,31,32,33]
Stop			
3′ UTR	See 5′ UTR (1 + 2) Early-on use of alpha globin 3′ UTR Tandem repeats of alpha globin 3′ UTR Optimal length is mandatory	Intracellular kinetics heavily depend on 3′ UTR Increase in protein synthesis during fibroblast conversion to induced pluripotent stem cells Critical for mRNA stability Too long: shorter half-life; too short: less efficient translation	See 5′ UTR [23,25,34,35,36]
Poly(A) tail	Length of the poly(A) tail, ideally > 90 A, shorter sequence is more efficient	Critical role for translation and stability	[20,37,38,39,40]

**Table 3:** Active ingredients and formulations of mRNA and viral-based COVID-19 vaccines approved in the European Union. Data have been retrieved from the European Public Assessment Report (EPAR). The structural modifications provided in Table 3 do not reflect all changes that have been introduced into the mRNA molecules; major portions are proprietary and have not been disclosed publicly.

Product	Active Ingredient	Formulation	Storage
Comirnaty, tozinameran, BNT162b2 *BioNTech* (Mainz, Germany)	Single-stranded, 5′-capped mRNA, encoding the spike antigen [glycoprotein (S)] of SARS-CoV-2 (isolate Wuhan-Hu-1) containing two consecutive proline mutations (P2 S); uridine substituted by *N*1-methylpseudouridine (1mΨ)	Multidose concentrate to be diluted prior to i.m. injection; dispersion of mRNA in LNPs containing ALC-0315 and ALC-0159 (functional lipids), DSPC and cholesterol (structural lipids) in aqueous cryoprotectant buffer.	−90 °C to −60 °C −25 °C to −15 °C (for 2 weeks)
Spikevax, elasomeran/imelasomeran (Omicron BA.1 variant) mRNA-1273 *Moderna*, (Camebridge, MA, USA)	Single-stranded, 5′-capped mRNA, encoding for the full-length SARS-CoV-2 spike protein modified with 2 proline substitutions within the heptad repeat 1 domain (S-2P); S protein composed of two subunits (S1 and S2) and stabilized in the pre-fusion conformation by two amino acid mutations, K986P and V987P; open reading frame of 3819 nucleotides; contains 1mΨ instead of uridine; undisclosed modification of the 5′ cap	Multidose dispersion for injection with mRNA encapsulated in lipid nanoparticles with the following main components: SM-102, cholesterol, DSPC, and PEG2000-DMG	−50 °C to −15 °C
Vaxzevria AZD1222 COVID-19 Vaccine (ChAdOx1-S [recombinant]) *AstraZeneca* (Cambridge, UK)	Single recombinant, replication-deficient chimpanzee adenovirus (ChAdOx1) vector expressing the S glycoprotein spike protein of SARS-CoV-2 with a tPA leader sequence; no mutations introduced in the expressed SARS-CoV-2 spike protein; non-encapsulated, icosahedral particles (virions of 80 to 100 nm diameter) containing a single copy of the double-stranded DNA genome	Liquid dosage form for i.m. injection	2 °C to 8 °C
COVID-19 Vaccine Ad26.COV2.S *Janssen* (Beerse, Belgium)	Recombinant, replication-incompetent adenovirus serotype 26 (Ad26) encoding the SARS-CoV-2 spike (S) protein	Liquid suspension containing 2-hydroxypropyl-β-cyclodextrin for i.m. injection	−25 °C to −15 °C
COVID-19 Vaccine (inactivated, adjuvanted) Valneva *Valneva Austria GmbH* (Wien, Austria)	Purified, inactivated, and adjuvanted whole virus SARS-CoV-2 (Italian strain (LAZ-INMI1-isl/2020, GISAID Accession number: EPI_ISL_410545)) vaccine grown on Vero cell culture	Liquid suspension for i.m. injection adjuvanted with hydrated aluminium hydroxide and CpG 1018 and recombinant human albumin produced in yeast	2 °C to 8 °C
Nuvaxovid NVX-CoV2373 COVID-19 vaccine (recombinant, adjuvanted) *Novavax CZ* (Jevany, Czechia)	Protein product of a recombinant SARS- CoV-2 S-gene (Wuhan-Hu-1) encoding the 1260 amino acid spike protein (the full-length 1273 amino acid protein minus the signal peptide); S gene codon optimized for expression in *Spodoptera frugiperda* (Sf9) insect cells; five amino acid changes introduced, including three in the S1/S2 furin cleavage site (RRAR to QQAQ) and two in the HR1 domain	Aqueous buffered dispersion for i.m. injection, co-formulated with Matrix-M1 adjuvant	2 °C to 8 °C

**Table 4:** Selected neoantigens for use in mRNA vaccinations.

Tumor Type	Neoantigen	Reference
Bladder cancer	AP2S1, P3H4, and RAC3	[174]
Melanoma	PTPRC, SIGLEC10, CARD11, LILRB1, and ADAMDEC1	[175]
Colorectal, NSCLC, and pancreatic cancers	KRAS	[176]
Esophageal squamous cell carcinoma (ESCC)	NLRC5, FCRL4, TMEM229B, and LCP2	[177]
Soft tissue sarcoma	HLTF, ITGA10, PLCG1, and TTC3	[178]
Glioblastoma	ADAMTSL4, COL6A1, CTSL, CYTH4, EGFLAM, LILRB2, MPZL2, SAA2, and LSP1	[179]
Glioma	NAT1, FRRS1, GTF2H2C, BRCA2, GRAP, NR5A2, ABCB4, ZNF90, ERCC6L, and ZNF813	[180]
Malignant mesothelioma	FAM134B, ALDH3A2, SAV1, RORC, and FN1	[181]
Stomach adenocarcinoma	ADAMTS18, COL10A1, PPEF1, and STRA6	[182]
Mesothelioma	AUNIP, FANCI, LASP1, PSMD8, and XPO5	[183]

**Table 5:** Selection of mRNAs in development for cancer treatment. FixVac: Non-mutated antigens shared among patients with a specific cancer type, applicable for almost all types of tumor antigens. iNeST: Targeting 20 neoantigens unique to each patient, applicable for almost all types of tumor antigens.

Name	mRNA	Indications	Admin.	Reference
BNT111 FixVac	4 TAAs: tyrosinase, NY-ESO-1, MAGE A3, TPTE	Advanced melanoma Phase I: Lipo-MERIT trial ± checkpoint inhibitor PD1 Phase II: + cemiplimab	Intravenous (i.v.) liposomal RNA (RNA-LPX)	[197] NCT02410733 NCT04526899
BNT112 FixVac	5 prostate cancer-specific antigens: kallikrein-2, kallikrein-3, acid phosphatase prostate, HOXB13, NK3 homeobox 1	Prostate cancer Phase I/II + cemiplimab PRO-MERIT trial	i.v. RNA-LPX	[198] NCT04382898
BNT113 FixVac	HPV16-E6 and -E7	HPV16^+^ head and neck cancer; AHEAD-MERIT Phase II + pembrolizumab HARE-40 Phase I/II	i.v. RNA-LPX	NCT04534205 NCT03418480
BNT115 W_ova1 Vaccine FixVac	3 ovarian cancer TAAs	Ovarian cancer Phase I + carboplatin/paclitaxel	i.v.	NCT04163094
BNT116 FixVac	6 mRNAs each of which encodes for a different TAA	NSCLC Phase I/II + cemiplimab Phase I + cemiplimab or docetaxel, LuCa-MERIT-1	i.v. Liposomes	NCT05557591 NCT05142189
BNT121 IVAC MUTANOME	Personalized vaccine	Metastatic melanoma Phase I ± RBL001/RBL002	Intranodal	[3,172] NCT02035956
BNT122 (RO7198457 autogene cevumeran) iNeST	20 patient-specific antigens	Multiple solid tumors Phase I	i.v.	[199] NCT03289962
		Melanoma Phase II + pembrolizumab	i.v.	NCT03815058
		NSCLC (adjuvant) Phase II + atezolizumab	i.v.	NCT04267237
		CRC Phase II	i.v.	NCT04486378
		Pancreatic cancer Phase I + atezolizumab + mFOLFIRINOX	i.v.	NCT04161755
BNT114 + BNT122 Personalized	IVAC_W_bre1_uID and IVAC_W_bre1_uID/IVAC_M_uID	Triple Negative Breast Cancer TNBC-MERIT	i.v.	NCT02316457
SAR441000 (BNT131)	IL-12sc, IL-15sushi, GM-CSF, IFNα	Solid tumors Phase I ± cemiplimab	Intratumoral	NCT03871348
BNT141	Encoded antibodies	Multiple solid tumors Phase I/II ± nab-paclitaxel and gemcitabine	i.v.	NCT04683939
BNT142	Encoded antibodies	Multiple solid CLDN6^+^ tumors Phase I/II	i.v.	NCT05262530
BNT151	Encoded cytokines: optimized IL-2	Multiple solid tumors (optimized IL-2) Phase I/II	i.v.	NCT04455620
BNT152	Encoded cytokines: IL-2, IL-7	Multiple solid tumors Phase I/II	i.v.	NCT04455620 NCT04710043
BNT153	Encoded cytokines: IL-2, IL-7	Multiple solid tumors Phase I	i.v.	NCT04710043
mRNA-2416	OX40L	Advanced malignancies Phase I/II ± durvalumab	Intratumoral LNP	[3] NCT03323398
mRNA-2752	OX40L, IL-23, IL-36γ	Advanced malignancies Phase I/II ± durvalumab	Intratumoral LNP	[200] NCT03739931
mRNA-4157 (V941)	Up to 34 neoantigens, personalized	High-risk melanoma, solid tumors KEYNOTE-603 Phase I + pembrolizumab KEYNOTE-942 Phase II + pembrolizumab	i.m. LNP	[201,202,203,204] NCT03313778 NCT03897881
mRNA-4650 NCI-4650	Up to 20 antigens + up to 15 HLA class I candidate neoantigens	Gastric or rectal cancer Phase I Melanoma Phase I/II	i.m.	[204,205] NCT03480152
mRNA-5671 Merck V941	4 KRAS mutations (G12D, G13D, G12C, and G12V), personalized	CRC, NSCLC, pancreatic adenocarcinoma Phase I ± pembrolizumab	i.m.	[204] NCT03948763
ECI-006	5 TAAs + 3 DC-activating antigens	Melanoma Phase I ± standard anti-PD-1	Intranodal TriMix	[204] NCT03394937
TriMix	3 mRNA encoding CD70, CD40L, and a constitutively active form of TLR4	Breast cancer Phase I	Intratumoral	NCT03788083
TriMixDC-MEL IPI	MAGE-A3, MAGE-C2, tyrosinase, and gp100	Melanoma Phase II + ipilimumab	Intratumoral	[206] NCT01302496
TriMix-DC		Melanoma Phase I Phase I/II	i.v. and intradermal	[207] NCT01066390
TriMix-DC + TLR-DC		Melanoma Phase I Phase I/II	i.v. (mRNA) intranodal (DCs)	[207] NCT01530698
TriMix: DC + mRNA (CD70, CD40) + TLR4	Tyrosinase, gp100, MAGE-A3, or MAGE-C2	Breast cancer Phase II + ipilimumab	Intratumoral	[206,207] NCT01302496
TriMixDC-MEL: Autologous monocyte-derived mRNA co-electroporated DCs + mRNA	CD40L, CD70, caTLR4	Melanoma Phase I	i.v.	[208]
CV8102	TLR7/8, RIG-1	Skin cancer Phase I	Intradermal Protamine	[209] NCT03291002
	TL	Hepatocellular carcinoma Phase I + IMA970A + cyclophosphamide	Intradermal Protamine	NCT03203005
CV9103 RNActive^®^	4 antigens for prostate cancer: PSA, PSMA, PSCA, STEAP	Prostate cancer Phase I/II Phase I/II	Intradermal	[210,211] NCT00906243 NCT00831467
CV9104 Mixture of 6 mRNAs, each encoding 1 antigen	PSA, PSCA, PSMA, STEAP1, PAP, MUC1	Prostate cancer Phase I/II Phase II	Intradermal or needle-free injection device (Tropis^®^, London, UK) Protamine	[210,212] NCT01817738 NCT02140138
CV9201	5 mRNAs: NY-ESO-1, MAGE C1, MAGE C2, survivin, TBG	NSCLC Phase I/II	Intradermal	[213] NCT00923312 NCT03164772
CV9202 BI 1361849	6 mRNAs encoding 6 different antigens: NY-ESO-1, MAGE C1, MAGE C2, TPBG, survivin, MUC1	NSCLC Phase I + local radiation Phase I/II ± durvalumab and tremelimumab	Intradermal	[214,215,216] NCT01915524 NCT03164772
MEDI1191	IL-12	Advanced solid tumors, prostate, breast cancer, NSCLC Phase I + durvalumab	Intratumoral LNP	[204,217] NCT03946800
Naked mRNA	Melan-A, MAGE-A1, MAGE-A3, survivin, GP100, and tyrosinase	Melanoma Phase I/II + GM-CSF Phase I/II + GM-CSF	Intradermal GM-CSF as adjuvant	[218] NCT00204516 NCT00204607
Naked mRNA	MUC1, CEA, Her-2/neu, telomerase, survivin, MAGE-A1	Renal cell cancer Phase I + durvalumab	Intradermal GM-CSF as adjuvant	[219]
SW1115C3	Cancer TSAs, personalized	Solid tumors Phase I	Subcutaneous	[209] NCT05198752
Tumor mRNA + pp65 flLAMP		Glioma, glioblastoma Phase I	i.v. RNA-LP (DOTAP liposome)	NCT04573140

## Abbreviations

1mΨ	N1-methyl-pseudouridine
ADAMDEC1	ADAM like decysin 1
ADAMTSL4	ADAMTS Like 4
ADAMTS18	ADAM metallopeptidase with thrombospondin type 1 motif 18
ALC-0159	2-[(polyethylene glycol)-2000]-*N*,*N*-ditetradecylacetamide
ALC-0315	((4-hydroxybutyl)azanediyl)bis(hexane-6,1-diyl)bis(2-hexyldecanoate)
ALDH3A2	aldehyde dehydrogenase 3 family member A2
ALL	acute lymphocytic leukemia
AP2S1	AP-2 complex subunit sigma
AUNIP	aurora kinase A and ninein interacting protein
BRAF	B-RAF, B rapidly accelerated fibrosarcoma
caTLR4	constitutively activated TLR4
CARD11	caspase recruitment domain family member 11
CEA	carcinoembryonic antigen
CLL	chronic lymphocytic leukemia
CMA	conditional marketing authorization
COL10A1	collagen type X alpha 1 chain
COL6A1	collagen type VI alpha 1 chain
COVID-19	coronavirus disease 2019
CpG 1018	synthetic oligomer cytosine phospho-guanine
CPI	checkpoint inhibitor
CRC	colorectal cancer
CTSL	cathepsin L
CYTH4	cytohesin 4
DC	dendritic cell
DLin-MC3-DMA	dilinoleylmethyl-4-dimethylaminobutyrate
DSPC	1,2-distearoyl-*sn*-glycero-3-phosphocholine
dsRNA	double-stranded RNA
DOPE	1,2-dioleoyl-*sn*-glycerol-3phosphoethanolamine
eIF4E	eukaryotic translation initiation factor 4E
EGFLAM	EGF-like, fibronectin type III and laminin G domains
ESCC	esophageal squameous cell carcinoma
FAM134B	family with sequence similarity 134, member B
FANCI	FA complementation group I
FCRL4	Fc receptor-like 4
flLAMP	full-length (fl) lysosomal-associated membrane protein (LAMP)
FN1	fibronectin 1
GM-CSF	granulocyte-macrophage colony-stimulating factor
Her-2/neu	human epidermal growth factor receptor 2
HLTF	helicase-like transcription factor
HBV	Hepatitis B
HOXB13	homeobox B13
HPLC	high-pressure liquid chromatography
HPV	human papillomavirus
IFN	interferon
ITGA10	integrin subunit alpha 10
IVT	in vitro transcription
KRAS	Kirsten rat sarcoma virus
LASP1	LIM And SH3 protein 1
LCP2	lymphocyte cytosolic protein 2
LILRB1	leukocyte immunoglobulin-like receptor B1
LSP1	lymphocyte-specific protein 1
MAGE-A1	melanoma-associated antigen family A1
MAGE-A3	melanoma antigen family A3
MAGE-C1	melanoma antigen family C1
MAGE-C2	melanoma antigen family C2
MERS	Middle East respiratory syndrome
MERS-CoV	Middle East respiratory syndrome-related coronavirus
MHC	major histocompatibility complex
MPZL2	Myelin protein zero like 2
mRNA	messenger ribonucleic acid
MDSC	myeloid-derived suppressor cell
MUC1	mucin 1
NHL	non-Hodgkin lymphoma
NLRC5	NOD(nucleotide-binding oligomerization)-like receptor family CARD domain containing 5
NRAS	neuroblastoma RAS
NSCLC	non-small cell lung cancer
NY-ESO-1	New York esophageal squamous cell carcinoma 1
ORF	open reading frame
OX40L	OX40 ligand
P3H4	prolyl 3-hydroxylase family member 4
PAP	phosphatidate phosphatase
PEG	polyethylene glycol
PEG2000-DMG	1,2-Dimyristoyl-rac-glycero-3-methoxypolyethylene glycol-2000
PKR	protein kinase R
PLCG1	phospholipase C gamma 1
Poly(A)	multiple adenosine units
PPEF1	protein phosphatase with EF- protein phosphatase with EF-hand domain 1
PPR	pentatricopeptide repeat
PSA	prostate-specific antigen
PSCA	prostate stem cell antigen
PSMA	prostate-specific membrane antigen
PSMD8	proteasome 26S subunit, non-ATPase 8
PTPRC	protein tyrosine phosphatase receptor type C
RAC3	Rac family small GTPase 3
RBD	receptor-binding domain
RIG-I	retinoic acid-inducible gene I
RORC	RAR-related orphan receptor C
SAA2	serum amyloid A2
saRNA	self-amplifying RNA
SARS	severe acute respiratory syndrome
SARS-CoV-2	severe acute respiratory syndrome coronavirus 2
SAV1	salvador family WW domain containing protein 1
SCLC	small cell lung cancer
SIGLEC10	sialic acid binding Ig like lectin 10
SM-102	(heptadecan-9-yl 8-{(2-hydroxyethyl)[6-oxo-6-(undecyloxy)hexyl]amino}octanoate)
STEAP1	six transmembrane epithelial antigen of the prostate 1
STRA6	signaling receptor and transporter of retinol STRA6
taRNA	trans-amplifying RNA
TAA	tumor-associated antigen
TPBG	trophoblast glycoprotein
TLR	toll-like receptor
TLR4	toll like receptor 4
TLR7	toll like receptor 7
TMEM229B	transmembrane protein 229B
TPTE	transmembrane phosphatase with tensin homology
TSA	tumor-specific antigen
TTC3	tetratricopeptide repeat domain 3
XPO5	exportin 5
